# Gene-Specific Differential DNA Methylation and Chronic Arsenic Exposure in an Epigenome-Wide Association Study of Adults in Bangladesh

**DOI:** 10.1289/ehp.1307884

**Published:** 2014-10-17

**Authors:** Maria Argos, Lin Chen, Farzana Jasmine, Lin Tong, Brandon L. Pierce, Shantanu Roy, Rachelle Paul-Brutus, Mary V. Gamble, Kristin N. Harper, Faruque Parvez, Mahfuzar Rahman, Muhammad Rakibuz-Zaman, Vesna Slavkovich, John A. Baron, Joseph H. Graziano, Muhammad G. Kibriya, Habibul Ahsan

**Affiliations:** 1 Department of Public Health Sciences, The University of Chicago, Chicago, Illinois, USA; 2 Department of Environmental Health Sciences, Mailman School of Public Health, Columbia University, New York, New York, USA; 3 U-Chicago Research Bangladesh, Dhaka, Bangladesh; 4 Department of Medicine, University of North Carolina School of Medicine, Chapel Hill, North Carolina, USA; 5 Department of Medicine, and; 6 Department of Human Genetics and Comprehensive Cancer Center, The University of Chicago, Chicago, Illinois, USA

## Abstract

Background: Inorganic arsenic is one of the most common naturally occurring contaminants found in the environment. Arsenic is associated with a number of health outcomes, with epigenetic modification suggested as a potential mechanism of toxicity.

Objective: Among a sample of 400 adult participants, we evaluated the association between arsenic exposure, as measured by blood and urinary total arsenic concentrations, and epigenome-wide white blood cell DNA methylation.

Methods: We used linear regression models to examine the associations between arsenic exposure and methylation at each CpG site, adjusted for sex, age, and batch. Differentially methylated loci were subsequently examined in relation to corresponding gene expression for functional evidence of gene regulation.

Results: In adjusted analyses, we observed four differentially methylated CpG sites with urinary total arsenic concentration and three differentially methylated CpG sites with blood arsenic concentration, based on the Bonferroni-corrected significance threshold of *p* < 1 × 10^–7^. Methylation of *PLA2G2C* (probe cg04605617) was the most significantly associated locus in relation to both urinary (*p* = 3.40 × 10^–11^) and blood arsenic concentrations (*p* = 1.48 × 10^–11^). Three additional novel methylation loci—*SQSTM1* (cg01225779), *SLC4A4* (cg06121226), and *IGH* (cg13651690)—were also significantly associated with arsenic exposure. Further, there was evidence of methylation-related gene regulation based on gene expression for a subset of differentially methylated loci.

Conclusions: We observed significant associations between arsenic exposure and gene-specific differential white blood cell DNA methylation, suggesting that epigenetic modifications may be an important pathway underlying arsenic toxicity. The specific differentially methylated loci identified may inform potential pathways for future interventions.

Citation: Argos M, Chen L, Jasmine F, Tong L, Pierce BL, Roy S, Paul-Brutus R, Gamble MV, Harper KN, Parvez F, Rahman M, Rakibuz-Zaman M, Slavkovich V, Baron JA, Graziano JH, Kibriya MG, Ahsan H. 2015. Gene-specific differential DNA methylation and chronic arsenic exposure in an epigenome-wide association study of adults in Bangladesh. Environ Health Perspect 123:64–71; http://dx.doi.org/10.1289/ehp.1307884

## Introduction

Millions of individuals worldwide are exposed to inorganic arsenic through drinking water as well as dietary sources (Smith et al. 2000). Arsenic is a well-established human carcinogen (International Agency for Research on Cancer 2012); however, the exact mechanism by which it causes cancer has not been established (Kitchin and Conolly 2010). There is *in vitro* and *in vivo* evidence to suggest that epigenetic alterations may mediate arsenic toxicity, as recently reviewed by Reichard and Puga (2010) and Ren et al. (2011).

Several human studies have examined global DNA methylation in blood in relation to arsenic exposure using surrogate markers of global DNA methylation, such as long interspersed nucleotide element-1 (LINE-1), Alu element methylation, methyl incorporation assays, or luminometric methylation assays. The findings from those studies have largely been inconsistent and included a number of differences in exposure measures and doses across studies (Hossain et al. 2012; Intarasunanont et al. 2012; Kile et al. 2012; Lambrou et al. 2012; Majumdar et al. 2010; Pilsner et al. 2007, 2009, 2012; Tajuddin et al. 2013; Wilhelm et al. 2010). Several other studies have evaluated arsenic in relation to gene-specific DNA methylation, most frequently assessing p16 and p53 promoter methylation (Chanda et al. 2006; Chen et al. 2007; Engström et al. 2013; Hossain et al. 2012; Intarasunanont et al. 2012; Marsit et al. 2006; Zhang et al. 2007). However, relatively few epigenome-wide DNA methylation studies have been conducted to investigate epigenetic alterations of arsenic toxicity in humans, evaluating associations with arsenical skin lesion status (Seow et al. 2014; Smeester et al. 2011), urinary arsenic species (Bailey et al. 2013), *in utero* arsenic exposure (Kile et al. 2014; Koestler et al. 2013a), toenail arsenic concentration (Liu et al. 2014), or arsenic-related urothelial carcinomas (Yang et al. 2014). Smeester et al. (2011) examined epigenome-wide promoter DNA methylation in peripheral blood leukocytes among 16 arsenic-exposed females from Mexico in relation to skin lesion status, and observed 183 differentially methylated genes, of which 182 were hypermethylated. Bailey et al. (2013) evaluated the data of Smeester et al. (2011) in relation to urinary arsenic species and observed nominally significant differential promoter DNA methylation in 812 unique genes, of which the majority were hypomethylated compared with relative urinary arsenic metabolite species. Koestler et al. (2013a) evaluated *in utero* arsenic exposure in relation to epigenome-wide cord blood methylation in 134 U.S.-based individuals and observed evidence of enrichment of hypermethylated loci in CpG islands, as well as a suggested indication of endocrine-disrupting effects of arsenic through hypomethylation of *ESR1* (estrogen receptor 1) and *PPARGC1A* (peroxisome proliferator-activated receptor gamma, coactivator 1 alpha).

The evidence noted above warrants further investigation of arsenic exposure on gene-specific DNA methylation in a comprehensive manner, and in a larger study population to identify potential mechanisms associated with arsenic-related toxicity. We conducted an epigenome-wide association study among 400 Bangladeshi individuals with manifest arsenical skin lesions to assess whether arsenic exposure level (as measured by blood arsenic and urinary total arsenic concentrations) is associated with differential white blood cell DNA methylation.

## Methods

*Study population*. The Bangladesh Vitamin E and Selenium Trial is a 2 × 2 factorial randomized chemoprevention trial evaluating the long-term effects of vitamin E and selenium supplementation on nonmelanoma skin cancer risk (Argos et al. 2013). Participants were residents of rural communities in central Bangladesh. Eligibility criteria included age between 25 and 65 years, permanent residence in the study area, manifest arsenical skin lesions, and no prior cancer history. Between April 2006 and August 2009, a total of 7,000 individuals were enrolled into the study. Trained study physicians, blinded to participants’ arsenic exposure, conducted in-person interviews and clinical evaluations, and collected urine and blood samples from participants in their homes using structured protocols. Of participants enrolled in the study, 413 were randomly sampled for epigenome-wide methylation analyses; baseline biological specimens collected before the start of the trial intervention were used in the analyses.

The study protocol was approved by the relevant institutional review boards in the United States (The University of Chicago and Columbia University) and Bangladesh (Bangladesh Medical Research Council). Informed consent was provided by participants prior to the baseline interview of the original study.

*Exposure assessment*. Urinary total arsenic concentration was measured in the baseline spot urine sample by graphite furnace atomic absorption spectrometry (AAnalyst 600 spectrometer; PerkinElmer, Norwalk, CT, USA) with a detection limit of 2 μg/L, in a single laboratory (Trace Metal Core Laboratory at Columbia University) (Nixon et al. 1991). Urinary creatinine was also measured for all participants in the same laboratory by a colorimetric method based on the Jaffe reaction (Heinegård and Tiderström 1973). Urinary total arsenic was divided by creatinine to obtain a creatinine-adjusted urinary total arsenic concentration, expressed as micrograms per gram creatinine. Creatinine-adjusted urinary total arsenic, a good biomarker of aggregate ingested arsenic exposure, captures exposure from all sources including water, food, soil, and dust (Hughes 2006).

Venous whole blood samples collected at baseline were analyzed for blood arsenic concentration by inductively coupled plasma mass spectrometry (ICP-MS) using a PerkinElmer Elan DRC (dynamic reaction cell) II equipped with an AS 93+ autosampler (PerkinElmer). ICP-MS-DRC methods for metals in whole blood were developed according to published procedures (Pruszkowski et al. 1998; Stroh 1988), with modifications for blood sample preparation as suggested by the Laboratory for ICP-MS Comparison Program (Institut National de Sante Publique du Québec).

*DNA methylation*. DNA was extracted using DNeasy Blood kits (Qiagen, Valencia, CA, USA), and bisulfite conversion was performed using the EZ DNA Methylation Kit (Zymo Research, Irvine, CA, USA). DNA methylation was measured in 500 ng of bisulfite-converted DNA per sample using the Illumina HumanMethylation 450K BeadChip kit (Illumina, San Diego, CA, USA) according to the manufacturer’s protocol; this beadchip allows interrogation of 485,577 CpG sites per sample. The methylation score for each CpG site, represented as the β value, on a continuous scale between 0 (unmethylated) and 1 (completely methylated) was quantile normalized. Among the 413 participants for whom DNA methylation data were generated, we excluded 6 samples for which the reported sex of the participant did not correspond with predicted sex based on methylation patterns of the X and Y chromosomes, and 7 samples with > 5% of CpGs either containing missing values or having *p* for detection > 0.05. This resulted in 400 samples retained for analyses. We omitted individual β values that were associated with a *p* for detection > 0.05. We also excluded probes on the X (*n* = 11,232) and Y (*n* = 416) chromosomes, probes with missing chromosome data (mostly control probes; *n* = 65), and probes with > 10% missing data across samples (*n* = 1,932); this resulted in a total of 471,932 probes included in the statistical analyses. Quantile-normalized β values were logit transformed and adjusted for batch variability using ComBat software (Johnson et al. 2007). Based on 11 samples run in duplicate across two different plates in these experiments, the average interassay Spearman correlation coefficient (*r*_s_) was 0.987 (range, 0.974–0.993).

*Gene expression*. Mononuclear cells were preserved in Buffer RLT and stored at –80°C; RNA was then extracted using the RNeasy Micro Kit from QIAGEN (Valencia, CA, USA). The concentration and quality of RNA was checked on a Nanodrop 1000 spectrophotometer (Thermo Scientific, Wilmington, DE, USA). cRNA synthesis was performed using 250 ng of RNA using the Illumina TotalPrep 96 RNA Amplification kit. Gene expression was measured using the Illumina HumanHT-12-v4 BeadChip utilizing 750 ng of cRNA according to the manufacturer’s protocol. The chip contains a total of 47,231 probes covering 31,335 genes. Quantile-normalized expression values were log_2_ transformed and adjusted for batch variability using ComBat software (Johnson et al. 2007). Gene expression data were available for the 400 individuals included in these analyses.

*Genotyping*. Genotyping procedures have been described in detail previously (Pierce et al. 2012). Briefly, DNA extraction was carried out from whole blood using the QIAamp 96 DNA Blood Kit (QIAGEN, Valencia, CA, USA). Any DNA sample with a concentration < 40 ng/μL, a 260 nm/280 nm ratio outside the range of < 1.6 to ≥ 2.1 (measured by Nanodrop 1000), or fragmented DNA < 2 kb (assessed by smearing in Agilent BioAnalyzer) was excluded. Genotyping was performed using the Illumina HumanCytoSNP-12 BeadChip utilizing 250 ng DNA according to the manufacturer’s protocol. Using 257,768 genotyped single nucleotide polymorphisms (SNPs) after quality control procedures, we performed imputation using MaCH on the basis of the HapMap 3 Gujarati Indians in Houston (GIH) population (Build 36; http://hapmap.ncbi.nlm.nih.gov/downloads/phasing/2009-02_phaseIII/HapMap3_r2/), yielding 1,211,988 SNPs after quality control procedures. Genotype data were available for 393 individuals included in these analyses.

*Replication study*. For replication of our top differentially methylated loci, we examined associations in an independent sample of 48 Bangladeshi adult males from the ongoing Folate and Creatinine Trial (FACT), prior to intervention. No participants in this sample had manifest arsenical skin lesions. The methods used to measure and analyze DNA methylation have been described in detail by Harper et al. (2013). Briefly, the water arsenic concentration was measured for all study participants at Columbia University, as previously described (Van Geen et al. 2002), and individuals were categorized as having low (50–100 μg/L, *n* = 25) or high (> 100 μg/L, *n* = 23) exposure for statistical analyses. After Ficoll separation, DNA from peripheral blood mononuclear cells (PBMCs) was extracted using the 5 PRIME ArchivePure DNA Blood Kit (Fisher Scientific, Pittsburgh, PA, USA). Epigenome-wide methylation of PBMC DNA was measured at the Roswell Park Cancer Institute (Buffalo, NY, USA) using the Illumina HumanMethylation 450K BeadChip kit. Data were processed using a standard quality control protocol and adjusted for batch effects prior to analyses using ComBat (Johnson et al. 2007). Here, we report associations from linear regression models.

*Statistical analyses*. For each CpG site, a separate linear regression model was run regressing the logit-transformed β value on continuous arsenic exposure (i.e., blood arsenic or urinary total arsenic concentration), sex, and age. Here, we present model coefficients and SEs from the linear regression models comparing the 75th versus the 25th percentile of each arsenic distribution. To correct for multiple comparisons, we considered a Bonferroni-corrected (*p* < 1 × 10^–7^) significance threshold; however, we present results for all loci with *p* < 1 × 10^–5^. For differentially methylated probes with *p* < 1 × 10^–5^, we used linear regression to examine the association of methylation with corresponding RNA transcript levels of the gene containing the methylation locus, adjusted for sex, age, and urinary total arsenic concentration. Methylation and expression data were adjusted for batch effects prior to analyses using ComBat (Johnson et al. 2007).

ComBat batch-adjusted methylation data were used to infer white blood cell type fractions using the statistical method of Houseman et al. (2012). Briefly, we used 466 methylation probes previously identified to be associated with leukocyte distributions (Houseman et al. 2012) to infer the distribution of white blood cell types in our study samples. Linear regression was used to evaluate the association between quartiles of arsenic exposure, based on the distribution in our study sample, in relation to the estimated cell type fractions adjusted for sex and age.

To evaluate previously reported associations from other studies, we conducted a lookup of individual CpG loci of interest within our data set. For these analyses a CpG locus associated with *p* < 0.05 was considered to be statistically significant. We used the R program MethLAB v1.5 (Kilaru et al. 2012) and SAS software (SAS Institute Inc., Cary, NC, USA) to run all analyses.

## Results

**Figure 1:**
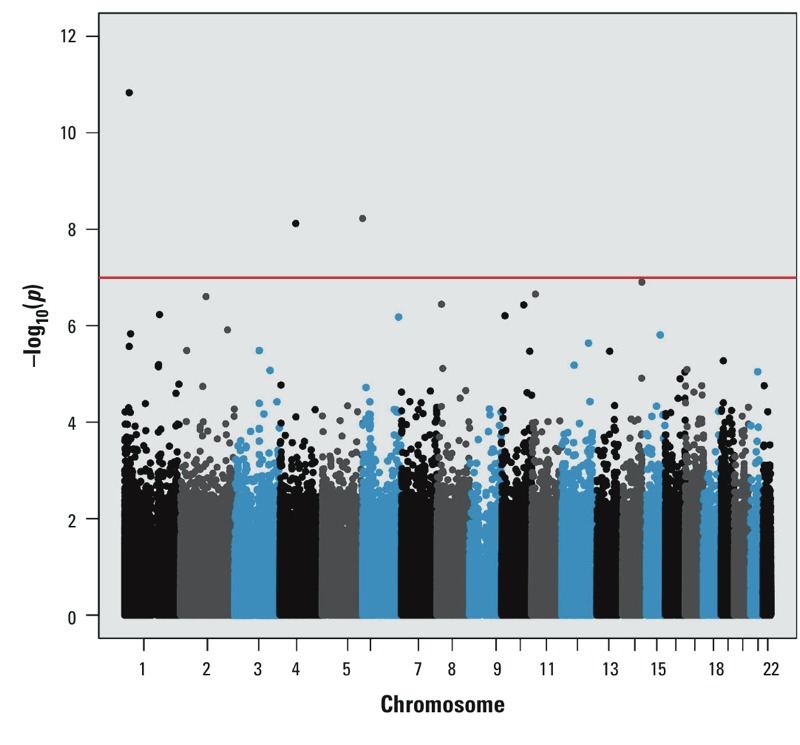
Manhattan plot for epigenome-wide association results for blood arsenic concentration. The horizontal red line corresponds to the significance threshold *p *= 1 × 10^–7^. Colors are used only to differentiate chromosomes.

**Figure 2:**
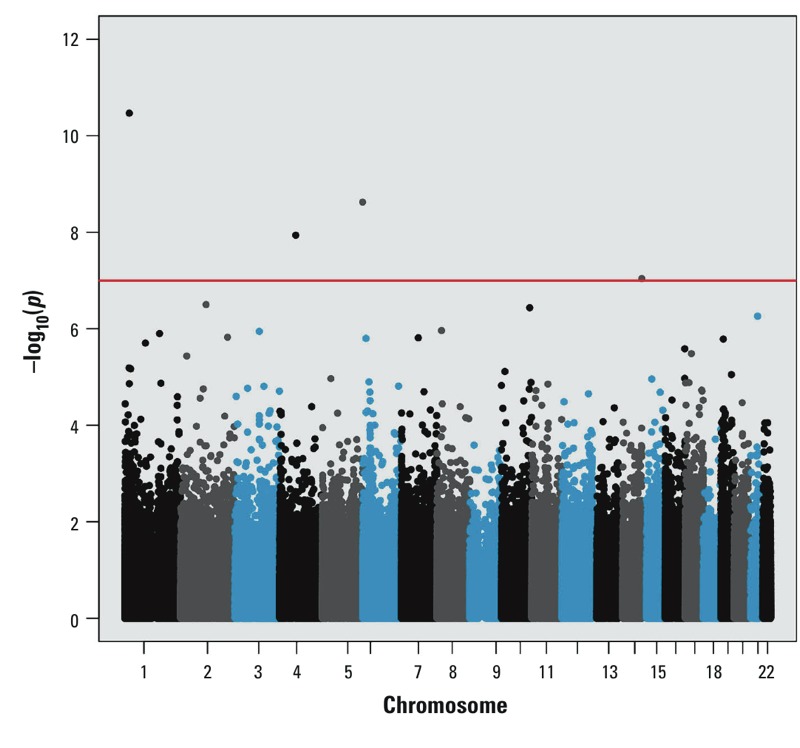
Manhattan plot for epigenome-wide association results for urinary total arsenic concentration. The horizontal red line corresponds to the significance threshold *p *= 1 × 10^–7^. Colors are used only to differentiate chromosomes.

Characteristics of the study sample are summarized in Table 1. We ran models separately for blood and urinary total arsenic concentrations (*r*_s_ = 0.91). We identified three loci that were significantly differentially methylated in relation to blood arsenic concentration based on the Bonferroni threshold *p* < 1 × 10^–7^, as shown in Figure 1. We identified four loci that were significantly differentially methylated in relation to urinary total arsenic concentration based on the Bonferroni threshold *p* < 1 × 10^–7^, as shown in Figure 2. The loci based on the Bonferroni threshold were common between the two analyses, and several overlapping associations that did not meet the strict Bonferroni threshold but that had *p* < 1 × 10^–5^ were also observed between the exposure analyses, as summarized in Table 2. Furthermore, there appeared to be enrichment for differentially methylated loci in relation to urinary and blood arsenic concentrations in ocean (isolated CpG loci in the genome) and CpG island shore regions (within 2 kb from a CpG island; see Supplemental Material, Figure S1). Only a single gene contained more than one differentially methylated locus with *p* < 1 × 10^–5^; hypermethylation of cg04605617 (chr1: 20,501,558) and cg08042135 (chr1: 20,501,758) in *PLA2G2C* was observed (*r*_s_ = 0.53). However, after adjustment of cg08042135 by cg04605617, the associations with blood (*p* = 0.14) and urinary total arsenic (*p* = 0.24) no longer persisted. Sensitivity analyses conducted with arsenic exposure as a natural log-transformed variable did not appreciably alter the association *p*-values (data not shown), suggesting robust linear associations for reported loci. In addition, the methylation β values are presented by urinary total arsenic exposure quartiles in Supplemental Material, Figure S2. In general, we observed dose-dependent trends between urinary total arsenic quartiles and DNA methylation levels. Box plots looked similar for blood arsenic quartiles (data not shown).

**Table 1:** Selected characteristics of the study sample.

Characteristic	*n* (%)
Sex	
Male	212 (53.0)
Female	188 (47.0)
Age (years)	43.3 ± 10.2^*a*^
25–35	104 (26.0)
36–45	130 (32.5)
46–55	122 (30.5)
56–65	44 (11.0)
Blood arsenic concentration (μg/L)	9.3 ± 11.3^*a*^
0.80–2.50	110 (27.7)
2.51–4.79	88 (22.2)
4.80–11.20	100 (25.2)
11.21–81.60	99 (24.9)
Urinary total arsenic concentration (μg/g)	302 ± 364.5^*a*^
12.0–75.0	97 (24.2)
75.1–139.9	105 (26.3)
140.0–394.9	98 (24.5)
395.0–2250.0	100 (25.0)
Values are *n* (%) unless otherwise noted. ^***a***^Mean ± SD.	

**Table 2:** Top 35 differentially methylated loci based on *p *< 1 × 10^–5^ in relation to blood or urinary total arsenic concentrations, sorted by chromosome (Chr).

CpG	Chr	Position	Gene	Feature category^*a*^	Median β value	Blood arsenic concentration			Urinary total arsenic concentration		
						Coefficient	SE	*p*-Value	Coefficient	SE	*p*-Value
cg02856716	1	18,993,307	*PAX7*	Body	0.102	–0.014	0.004	2.28 × 10^–4^	–0.019	0.004	6.46 × 10^–6^
cg04605617^*b*^	1	20,501,558	*PLA2G2C*	1st exon	0.724	0.049	0.007	1.48 × 10^–11^*	0.054	0.008	3.40 × 10^–11^*
cg08042135	1	20,501,758	*PLA2G2C*	TSS200	0.774	0.019	0.004	2.71 × 10^–6^	0.020	0.004	1.37 × 10^–5^
cg13223043	1	26,492,308	*FAM110D||ZNF593*	Intergenic	0.448	–0.021	0.004	1.48 × 10^–6^	–0.022	0.005	6.78 × 10^–6^
cg00857921	1	92,257,380	*TGFBR3*	Body	0.393	0.016	0.004	4.12 × 10^–5^	0.021	0.004	1.98 × 10^–6^
cg19750321	1	150,808,974	*ARNT*	Body	0.853	0.017	0.004	7.06 × 10^–6^	0.016	0.004	2.20 × 10^–4^
cg03984502	1	151,805,662	*RORC*	TSS1500	0.816	0.020	0.004	6.44 × 10^–6^	0.015	0.005	2.34 × 10^–3^
cg07207669	1	155,102,389	*EFNA1*	Body	0.560	–0.023	0.005	5.90 × 10^–7^	–0.025	0.005	1.27 × 10^–6^
cg08438392	2	27,708,645	*IFT172*	Body	0.782	0.020	0.004	3.27 × 10^–6^	0.022	0.005	3.66 × 10^–6^
cg00522451	2	113,464,049	*SLC20A1||NT5DC4*	Intergenic	0.216	–0.025	0.005	2.49 × 10^–7^	–0.028	0.005	3.16 × 10^–7^
cg00281776^*c*^	2	209,224,226	*PIKFYVE||PTH2R*	Intergenic	0.817	–0.038	0.008	1.22 × 10^–6^	–0.042	0.009	1.50 × 10^–6^
cg25881170	3	107,810,508	*CD47*	TSS1500	0.473	0.019	0.004	3.28 × 10^–6^	0.022	0.005	1.14 × 10^–6^
cg24262469	3	156,391,694	*TIPARP-AS1*	Body	0.371	0.022	0.005	8.48 × 10^–6^	0.022	0.005	4.98 × 10^–5^
cg06121226	4	72,134,061	*SLC4A4*	Body	0.463	–0.054	0.009	7.63 × 10^–9^*	–0.059	0.010	1.16 × 10^–8^*
cg01225779	5	179,238,473	*SQSTM1*	5’ UTR	0.562	–0.042	0.007	6.00 × 10^–9^*	–0.048	0.008	2.37 × 10^–9^*
cg19937878	6	13,296,150	*TBC1D7*	Body	0.820	0.028	0.007	1.91 × 10^–5^	0.035	0.007	1.59 × 10^–6^
cg11230112^*d*^	6	158,460,980	*SYNJ2*	Body	0.872	–0.029	0.006	6.60 × 10^–7^	–0.029	0.007	1.54 × 10^–5^
cg22813794	7	75,677,469	*MDH2*	TSS200; 5’ UTR	0.039	–0.059	0.014	5.49 × 10^–5^	–0.077	0.016	1.54 × 10^–6^
cg15310871	8	20,077,937	*ATP6V1B2*	3’ UTR	0.825	–0.039	0.008	3.61 × 10^–7^	–0.042	0.008	1.09 × 10^–6^
cg22977892	8	25,907,769	*PPP2R2A*	Body	0.254	0.021	0.005	7.70 × 10^–6^	0.020	0.005	1.20 × 10^–4^
cg02268561	10	15,212,066	*NMT2*	TSS1500	0.118	–0.026	0.005	6.23 × 10^–7^	–0.026	0.006	7.74 × 10^–6^
cg15108641	10	99,263,321	*UBTD1*	Body	0.365	0.033	0.006	3.70 × 10^–7^	0.030	0.007	3.11 × 10^–5^
cg07545081	10	126,308,381	*FAM53B*	3’ UTR	0.685	0.021	0.005	3.42 × 10^–6^	0.026	0.005	3.68 × 10^–7^
cg02742555	11	15,200,693	*INSC*	Body	0.163	–0.028	0.005	2.22 × 10^–7^	–0.026	0.006	2.74 × 10^–5^
cg03348792	12	53,075,482	*KRT1*	TSS1500	0.309	0.019	0.004	6.62 × 10^–6^	0.019	0.005	8.91 × 10^–5^
cg06383241	12	116,997,023	*MAP1LC3B2*	TSS200	0.083	–0.022	0.005	2.29 × 10^–6^	–0.022	0.005	2.23 × 10^–5^
cg11582226	13	77,587,297	*FBXL3*	Body	0.756	0.021	0.004	3.40 × 10^–6^	0.019	0.005	1.98 × 10^–4^
cg13651690	14	106,320,748	*IGH*	Body	0.950	0.035	0.006	1.24 × 10^–7^	0.039	0.007	9.16 × 10^–8^*
cg05018460	15	80,688,079	*AB240015||ARNT2*	Intergenic	0.557	0.047	0.010	1.56 × 10^–6^	0.046	0.011	2.09 × 10^–5^
cg04352288^*e*^	16	87,958,408	*CA5A*	Body	0.872	0.047	0.011	9.20 × 10^–6^	0.056	0.012	2.60 × 10^–6^
cg17892169	17	7,452,644	*TNFSF12-TNFSF13*	Body	0.341	0.024	0.005	8.06 × 10^–6^	0.023	0.006	2.01 × 10^–4^
cg08285388	17	27,230,177	*DHRS13*	TSS200	0.090	–0.014	0.004	1.37 × 10^–3^	–0.022	0.005	3.29 × 10^–6^
cg13480898	19	10,195,915	*C19orf66*	TSS1500	0.680	–0.023	0.005	5.35 × 10^–6^	–0.026	0.005	1.63 × 10^–6^
cg06381803	19	46,119,476	*EML2*	Body	0.400	–0.042	0.010	5.72 × 10^–5^	–0.051	0.011	8.95 × 10^–6^
cg26390598	21	41,032,397	*B3GALT5*	5’ UTR	0.340	0.050	0.011	9.03 × 10^–6^	0.063	0.012	5.49 × 10^–7^
Abbreviations: TSS, transcription start site; TSS200, 200 bases from TSS; TSS1500, 1,500 bases from TSS; UTR, untranslated region. “||” indicates an intergenic region. Four probes contain SNPs with a minor allele frequency ≥ 0.01. All SNPs > 10 bases from query site. ^***a***^Gene feature category of the methylation locus. ^***b***^rs12139100. ^***c***^rs139141387. ^***d***^rs73795212. ^***e***^rs113904153. *p < 1 × 10^–7^.											

Among all 471,932 CpG loci evaluated, 56.6% of the methylation probes were hypermethylated (*t*-statistic > 0) and 43.4% were hypomethylated (*t*-statistic < 0) in relation to urinary total arsenic concentration. Results were similar in relation to blood arsenic concentration (data not shown). Among the top 35 differentially methylated loci, blood and urinary total arsenic levels were associated with both gene-specific DNA hypermethylation (*n* = 19; 54.3%) and gene-specific DNA hypomethylation (*n* = 16; 45.7%), as shown in Table 2 (*p* = 0.78 for enrichment). Furthermore, no statistically significant association of arsenic exposure was observed in relation to global methylation levels across autosomes on a genomic scale. In a global analysis evaluating the association between arsenic exposure and average β values across all available CpG sites, we observed no significant global methylation patterns for blood (*p* = 0.124) or urinary total arsenic (*p* = 0.241) concentrations.

Among the 35 differentially methylated loci with *p* < 1 × 10^–5^, 29 loci could be evaluated in the replication sample. Of these, 8 methylation loci were associated with *p* < 0.05, with 5 loci observed to have the same direction of effect as the discovery association (see Supplemental Material, Table S1). The strongest replication signal was observed for *IGH* (cg13651690; *p* = 5.40 × 10^–3^). The Kolmogorov test indicated that the replication *p*-values were significantly different from a uniform *p*-value distribution (*p* = 0.0011).

Methylation probes containing known SNPs were not removed from analyses *a priori* because genome-wide SNP data were available for the study sample (Pierce et al. 2012) and could be examined in stratified analyses. As shown in Table 2, among the significantly differentially methylated loci based on Bonferroni criteria, one probe contained a SNP with minor allele frequency ≥ 0.01. Methylation probe cg04605617 contained SNP rs12139100 for which genotype data were available on 363 of the 400 study participants. The A allele frequency in the study sample was 0.38. In stratified analyses, the association between blood and urinary total arsenic concentrations in relation to DNA methylation at cg04605617 was observed to be independent of the rs12139100 genotype. Among individuals with the GG genotype (*n* = 136), associations persisted in relation to DNA methylation for blood arsenic concentration (*p* = 3.08 × 10^–4^) and urinary total arsenic concentration (*p* = 6.20 × 10^–5^), as well as among individuals with the GA+AA genotype (*n* = 227) for blood arsenic concentration (*p* = 1.61 × 10^–7^) and urinary total arsenic concentration (*p* = 2.43 × 10^–7^).

We examined the correlation between white blood cell DNA methylation with corresponding PBMC gene expression for the top 35 differentially methylated probes among the 400 study participants. Gene expression signals based on RNA transcripts for the corresponding genes containing the differentially methylated loci are summarized in Table 3. Among the 35 differentially methylated loci with *p* < 1 × 10^–5^, we could evaluate corresponding RNA transcript levels from the same gene for 28 methylation loci. Of these, 15 methylation loci were significantly associated with gene expression based on *p* < 0.1.

**Table 3:** Top 35 differentially methylated loci based on *p *< 1 × 10^–5^ in relation to peripheral blood mononuclear cell (PMBC) gene expression, sorted by chromosome (Chr).

CpG	Chr	Position	Gene	Feature category^*a*^	Expression probe	Probe coordinates	*p*-Value	Direction
cg02856716	1	18,993,307	*PAX7*	Body	ILMN_1835658	19,074,941–19,074,990	0.574	+
					ILMN_1761061	19,062,405–19,062,454	0.818	+
cg04605617	1	20,501,558	*PLA2G2C*	1st Exon	ILMN_3237030	20,501,573–20,501,622	0.073	+
					ILMN_1656867	20,490,548–20,490,597	0.324	–
cg08042135	1	20,501,758	*PLA2G2C*	TSS200	ILMN_1656867	20,490,548–20,490,597	0.742	–
					ILMN_3237030	20,501,573–20,501,622	0.758	+
cg13223043	1	26,492,308	*FAM110D||ZNF593*	Intergenic	NA
cg00857921	1	92,257,380	*TGFBR3*	Body	ILMN_1784287	92,148,163–92,148,212	1.620 × 10^–8^	–
cg19750321	1	150,808,974	*ARNT*	Body	ILMN_1762582	150,782,288–150,782,337	0.351	+
					ILMN_2347314	150,782,989–150,783,038	0.552	–
cg03984502	1	151,805,662	*RORC*	TSS1500	ILMN_1771126	151,778,991–151,779,040	3.001 × 10^–6^	–
					ILMN_1734366	151,778,901–151,778,950	4.770 × 10^–6^	–
					ILMN_1651792	151,804,215–151,804,264	0.247	+
					ILMN_2275399	151,798,406–151,798,455	0.992	–
cg07207669	1	155,102,389	*EFNA1*	Body	ILMN_2371055	155,107,215–155,107,264	0.140	+
					ILMN_2371053	155,106,804–155,106,853	0.272	–
cg08438392	2	27,708,645	*IFT172*	Body	ILMN_1784178	27,668,277–27,668,621	0.428	–
cg00522451	2	113,464,049	*SLC20A1||NT5DC4*	Intergenic	NA
cg00281776	2	209,224,226	*PIKFYVE||PTH2R*	Intergenic	NA
cg25881170	3	107,810,508	*CD47*	TSS1500	ILMN_2356991	107,762,694–107,762,743	0.044	+
					ILMN_1771333	107,762,387–107,762,436	0.060	+
cg24262469	3	156,391,694	*TIPARP-AS1*	Body	ILMN_3239662	157,875,849–157,875,878	0.223	+
cg06121226	4	72,134,061	*SLC4A4*	Body	ILMN_1734897	72,437,058–72,437,107	2.173 × 10^–4^	+
					ILMN_2184556	72,437,334–72,437,383	0.029	+
cg01225779	5	179,238,473	*SQSTM1*	5’ UTR	NA
cg19937878	6	13,296,150	*TBC1D7*	Body	ILMN_1661622	13,305,354–13,305,403	0.567	–
cg11230112	6	158,460,980	*SYNJ2*	Body	ILMN_2215119	158,438,833–158,438,882	0.752	+
cg22813794	7	75,677,469	*MDH2*	TSS200; 5’ UTR	ILMN_2079004	75,695,852–75,695,901	0.867	+
cg15310871	8	20,077,937	*ATP6V1B2*	3’ UTR	ILMN_1787705	20,078,945–20,078,994	0.004	–
cg22977892	8	25,907,769	*PPP2R2A*	Body	NA
cg02268561	10	15,212,066	*NMT2*	TSS1500	ILMN_2062620	15,147,945–15,147,994	0.030	+
cg15108641	10	99,263,321	*UBTD1*	Body	ILMN_1794914	99,330,592–99,330,641	2.416 × 10^–6^	–
cg07545081	10	126,308,381	*FAM53B*	3’ UTR	ILMN_2053490	126,308,101–126,308,150	0.280	–
					ILMN_1704571	126,308,260–126,308,309	0.834	–
cg02742555	11	15,200,693	*INSC*	Body	ILMN_1756070	15,262,019–15,262,068	0.343	+
					ILMN_2340643	15,267,549–15,267,598	0.983	+
cg03348792	12	53,075,482	*KRT1*	TSS1500	ILMN_1735712	53,068,865–53,068,914	0.298	+
cg06383241	12	116,997,023	*MAP1LC3B2*	TSS200	ILMN_3247613	115,481,580–115,481,621	0.005	–
cg11582226	13	77,587,297	*FBXL3*	Body	ILMN_2071405	77,579,718–77,579,767	0.299	+
cg13651690	14	106,320,748	*IGH*	Body	NA
cg05018460	15	80,688,079	*AB240015||ARNT2*	Intergenic	NA
cg04352288	16	87,958,408	*CA5A*	Body	ILMN_1731292	87,921,734–87,921,783	0.004	+
cg17892169	17	7,452,644	*TNFSF12-TNFSF13*	Body	ILMN_1683700	7,460,967–7,461,016	0.022	–
					ILMN_2399190	7,462,480–7,462,529	0.031	+
					ILMN_1784264	7,464,109–7,464,339	0.150	+
					ILMN_1670188	7,452,806–7,453,453	0.335	–
					ILMN_1680003	7,457,089–7,457,138	0.819	+
cg08285388	17	27,230,177	*DHRS13*	TSS200	ILMN_1790781	27,224,816–27,224,865	0.366	–
cg13480898	19	10,195,915	*C19orf66*	TSS1500	ILMN_1750400	10,203,832–10,203,881	0.004	+
cg06381803	19	46,119,476	*EML2*	Body	ILMN_3240541	50,804,842–50,804,886	0.028	–
cg26390598	21	41,032,397	*B3GALT5*	5’ UTR	ILMN_1698756	39,954,012–39,954,061	0.059	–
					ILMN_2378654	39,956,034–39,956,083	0.206	–
					ILMN_1800713	39,956,436–39,956,485	0.376	–
Abbreviations: –, inverse association between methylation and gene expression levels; +, positive association between methylation and gene expression levels; NA, not available; TSS, transcription start site; TSS200, 200 bases from TSS; TSS1500, 1,500 bases from TSS; UTR, untranslated region. “||” indicates an intergenic region. ^***a***^Gene feature category of the methylation locus.								

To evaluate the potential effect of arsenic exposure on white blood cell type proportions, we utilized estimated cell type fractions based on a validated subset of the methylation data, presented by quartiles of arsenic exposure in Table 4. We observed no notable effect of arsenic on cell type proportions, except for moderate estimated percent decreases in CD4^+^ T cells and natural killer (NK) cells in relation to the highest quartile of urinary total arsenic concentration as well as borderline associations (*p* = 0.13–0.14) in relation to the highest quartile of blood arsenic concentration.

**Table 4:** Estimated change in leukocyte cell type proportions by blood and urinary total arsenic concentrations.

Exposure	CD8^+^ T cells	CD4^+^ T cells	NK cells	B cells	Monocytes	Granulocytes
Blood arsenic concentration (μg/L)
0.80–2.50	Reference	Reference	Reference	Reference	Reference	Reference
2.51–4.79	0.39 (–1.09, 1.87)	–0.57 (–1.59, 0.44)	0.05 (–0.06, 0.15)	–0.46 (–1.18, 0.26)	0.04 (–0.47, 0.55)	0.55 (–1.16, 2.26)
4.80–11.20	1.02 (–0.43, 2.47)	–0.30 (–1.29, 0.70)	0.02 (–0.08, 0.12)	–0.39 (–1.09, 0.32)	–0.05 (–0.54, 0.45)	–0.31 (–1.99, 1.37)
11.21–81.60	0.47 (–0.97, 1.90)	–0.75 (–1.74, 0.24)	–0.08 (–0.18, 0.02)	–0.41 (–1.11, 0.29)	0.15 (–0.34, 0.64)	0.61 (–1.05, 2.28)
Urinary total arsenic concentration (μg/g)
12.0–75.0	Reference	Reference	Reference	Reference	Reference	Reference
75.1–139.9	0.37 (–1.08, 1.81)	–0.72 (–1.71, 0.28)	–0.08 (–0.18, 0.02)	–0.31 (–1.01, 0.39)	0.25 (–0.24, 0.75)	0.49 (–1.18, 2.16)
140.0–394.9	1.21 (–0.27, 2.69)	–0.47 (–1.50, 0.55)	–0.02 (–0.13, 0.08)	–0.35 (–1.07, 0.37)	0.20 (–0.31, 0.70)	–0.56 (–2.28, 1.15)
395.0–2250.0	0.99 (–0.47, 2.45)	–1.18 (–2.19, –0.17)*	–0.12 (–0.22, –0.01)*	–0.68 (–1.39, 0.03)	0.18 (–0.32, 0.68)	0.81 (–0.88, 2.50)
*p < 0.05.						

## Discussion

The findings of this study suggest associations of blood and urinary total arsenic concentrations with gene-specific DNA methylation changes. We identified four novel methylation loci in *PLA2G2C*, *SQSTM1*, *SLC4A4*, and *IGH* that were strongly associated with arsenic exposure (*p* < 1 × 10^–7^), as well as several suggestive associations in other gene regions. In addition, we observed that several of the differentially methylated loci were associated with corresponding gene expression levels in PBMCs.

Higher arsenic exposure was associated with increased methylation levels at cg04605617 (chr1: 20,501,558), located in the first exon of *PLA2G2C*. This locus was moderately associated with increased gene expression of *PLA2G2C* (ILMN_3237030, *p* = 0.073). *PLA2G2C* encodes a calcium-dependent phospholipase, which is an enzyme involved in the hydrolysis of phospholipids into free fatty acids and lysophospholipids. These lipid mediators have diverse biological functions relevant for cancer progression, including roles in inflammation and cell growth, signaling, and death (Dennis et al. 2011; Scott et al. 2010). Notably, phospholipase A2 enzymes have been shown to be induced by skin carcinogens (e.g., phorbol ester, ultraviolet B light), which ultimately leads to prostaglandin synthesis via cyclooxygenase-2 (COX2) leading to increased keratinocyte proliferation and skin carcinogenesis (Bowden 2004; Kast et al. 1993). Arsenic is an established skin carcinogen, with evidence from animal studies suggesting overexpression of COX2 associated with arsenic exposure (Ouyang et al. 2007; Tokar et al. 2011; Trouba and Germolec 2004).

Higher arsenic exposure was associated with decreased methylation levels at the cg01225779 locus (chr5: 179,238,473), located in the 5´ untranslated region (UTR) of *SQSTM1*. *SQSTM1* encodes a protein that binds ubiquitin and regulates activation of the nuclear factor kappa-B (NF-κB) signaling pathway. *SQSTM1* has been implicated in a number of diseases including neurodegenerative diseases, cancer, obesity, and insulin resistance (Geetha et al. 2012). *In vitro* studies have shown that arsenic induces the NF-κB pathway and may be a potential mechanism for skin carcinogenesis (Liao et al. 2004; Zuo et al. 2012).

Higher arsenic exposure was associated with decreased methylation levels at the cg06121226 locus (chr4: 72,134,061). This locus, located in the body of *SLC4A4*, was strongly associated with increased gene expression of *SLC4A4* (ILMN_2356991, *p* = 2.173 × 10^–4^ and ILMN_2184556, *p* = 0.029). *SLC4A4* encodes a sodium bicarbonate cotransporter involved in the regulation of bicarbonate secretion and absorption, as well as intracellular pH. Mutations in this gene have been associated with hypertension (Yang et al. 2012), a well-established health outcome associated with arsenic exposure (Abhyankar et al. 2012).

Higher arsenic exposure was also associated with increased methylation levels at the cg13651690 locus (chr14: 106,320,748), located in the body of *IGH*. The immunoglobulin heavy locus includes variable (V), diversity (D), joining (J), and constant (C) segments of immunoglobulins. Translocations in this region have been implicated in lymphoma (Guais et al. 2004). Arsenic exposure has been associated with elevated serum immunoglobulins (Islam et al. 2007), which may be involved in skin carcinogenesis (Wiemels et al. 2011). Furthermore, we observed significant replication of this locus in an independent study sample in relation to water arsenic concentration.

The biological implications of these findings must be further explored with regard to their role in various mechanisms of arsenic toxicity and arsenic-related disease outcomes. Chronic exposure to arsenic in drinking water has been associated with a multitude of health effects, including increased risks of cancer, cardiovascular disease, peripheral neuropathy, and respiratory diseases (Brouwer et al. 1992; Chen et al. 1992; Milton and Rahman 2002; Navas-Acien et al. 2005), as well as a possible association with diabetes (Argos et al. 2013; Navas-Acien et al. 2006). Future studies should be designed to evaluate phenotype-specific methylation patterns in arsenic-exposed populations. In addition, we found that the magnitude of differential methylation at each locus associated with arsenic exposure was small, although similar in size to estimated effects previously reported for arsenic and other environmental exposures (Joubert et al. 2012; Koestler et al. 2013a). The biological implications of relatively small changes in DNA methylation need to be elucidated further.

Among the seven epigenome-wide studies regarding arsenic-related traits published to date (Bailey et al. 2013; Kile et al. 2014; Koestler et al. 2013a; Liu et al. 2014; Seow et al. 2014; Smeester et al. 2011; Yang et al. 2014), only two of the studies did not use the Illumina platform for measurement of DNA methylation (Bailey et al. 2013; Smeester et al. 2011). In the present study, we observed no overlap between our top 35 differentially methylated loci and the top signals previously reported for other arsenic-related traits among studies that used the Illumina methylation array, although none of these prior studies identified statistically differentially methylated loci based on a Bonferroni threshold (*p* < 1 × 10^–7^). We also conducted a lookup of the top reported CpG signals from the previously published studies in our data set (see Supplemental Material, Table S2). Nominally significant associations in our data set have previously been reported for *AGAP2* cg11511175 and *RHBDF1* cg03333116 in relation to arsenical skin lesion status (Seow et al. 2014); *ELL* cg22489759 and *SNRNP200* cg00088989 in relation to toenail arsenic concentration (Liu et al. 2014); and *RIN2* cg03512414, *SLC12A6* cg11293029, *CBFA2T3* cg09051215, and *CCDC73* cg01717164 in relation to *in utero* arsenic exposure (Koestler et al. 2013a).

Furthermore, we evaluated the methylation of genes that were previously reported in candidate promoter methylation studies (Banerjee et al. 2013; Chanda et al. 2013; Gribble et al. 2014; Hossain et al. 2012; Intarasunanont et al. 2012) using a lookup approach in our data set for *DAPK1*, *CDKN2A* (*P16*), *GMDS*, *C10orf32*/*AS3MT*, *RASSF1*, *PPARG*, *TP53*, and *MLH1* (see Supplemental Material, Table S3). Our data provide supporting evidence for differential methylation specifically in the promoter regions of *CDKN2A* (cg03079681), *RASSF1* (cg06117233), *TP53* (cg05479194, cg02855142, cg08119584, and cg01620719), and *MLH1* (cg11291081 and cg05670953) in relation to arsenic exposure (see Supplemental Material, Table S3). Given the notable differences in arsenic toxicity constructs, arsenic exposure levels, and participant populations across published studies, future research is needed to further synthesize the existing evidence and elucidate the role of epigenetic mechanisms in relation to arsenic exposure and related diseases.

This study has several potential limitations. We measured DNA methylation in total white blood cells, which comprise various leukocyte subtypes known to be associated with differential methylation signatures (Adalsteinsson et al. 2012; Reinius et al. 2012). If arsenic exposure was associated with a substantial shift in leukocyte subtypes, then our analyses of DNA methylation in white blood cells may be confounded due to differences in cell type proportions. Because frozen unfractioned blood was used in these experiments, we could not evaluate the association between arsenic and cell type fractions directly. Therefore, we utilized a novel statistical method to infer expected cell type fractions in our study samples based on a validated subset of methylation markers as a surrogate measure (Houseman et al. 2012); the assumptions of the statistical method have been described elsewhere (Koestler et al. 2013a, 2013b). Based on these analyses, arsenic did not appear to be strongly associated with cell-type shifts, except for associations of the highest quartile of urinary total arsenic concentration with decreased CD4^+^ T and NK cell fractions. Therefore, we do not believe that the results observed in our analyses can be fully explained through an immunotoxic pathway of arsenic.

Another potential consideration for the findings of our study is that all participants had manifest arsenic skin lesions, which is a proxy for both chronic arsenic exposure and genetic susceptibility to arsenic toxicity. Because genotype is known to influence DNA methylation patterns (Tycko 2010), it is possible that the associations observed in our study may not be generalizable to populations without skin lesions. However, individuals with arsenical skin lesions are at increased risk of developing arsenic-related cancers and other disease conditions; therefore, the results of this study offer valuable insight into potential mechanistic pathways related to arsenic toxicity and carcinogenesis. Another potential limitation is that we did not validate the methylation signals identified with the Illumina platform using additional confirmatory methods. Because previously published studies have indicated very good concordance of the Illumina 450K platform with pyrosequencing data (Roessler et al. 2012), we did not pursue validation methods.

The major strengths of the present study are the relatively large size of the study sample, the multiple measures of arsenic exposure, the broad exposure range, and the availability of epigenome-wide methylation data, as well as genome-wide expression and genetic data from the study sample. Whereas previous studies have demonstrated an association between arsenic exposure and DNA methylation, we were also able to evaluate potentially functional gene regulation associated with the differentially methylated loci.

## Conclusions

Arsenic exposure was associated with differential gene-specific white blood cell DNA methylation at several novel loci. We also observed functional evidence of gene deregulation that corresponded with differential methylation at a subset of these loci. The clinical implications of these findings in arsenic-exposed populations require further investigation.

## Supplemental Material

(1.2 MB) PDF
