# Supplementary material for: Gene-Specific Differential DNA Methylation and Chronic Arsenic Exposure in an Epigenome-Wide Association Study of Adults in Bangladesh
Source: Environ Health Perspect. 2014 Oct 17;123(1):64–71. doi: 10.1289/ehp.1307884 (PMC4286273; doi:10.1289/ehp.1307884)
Supplement: (1.2 MB) PDF [file ehp.1307884.s001.508.pdf]

Environmental Health Perspectives doi:10.1289/ehp.1307884

Supplemental Material

Gene-Specific Differential DNA Methylation and Chronic Arsenic Exposure in an Epigenome-Wide Association Study of Adults in Bangladesh

Maria Argos, Lin Chen, Farzana Jasmine, Lin Tong, Brandon L. Pierce, Shantanu Roy, Rachelle Paul-Brutus, Mary V. Gamble, Kristin N. Harper, Faruque Parvez, Mahfuzar Rahman, Muhammad Rakibuz-Zaman, Vesna Slavkovich, John A. Baron, Joseph H. Graziano, Muhammad G. Kibriya, and Habibul Ahsan

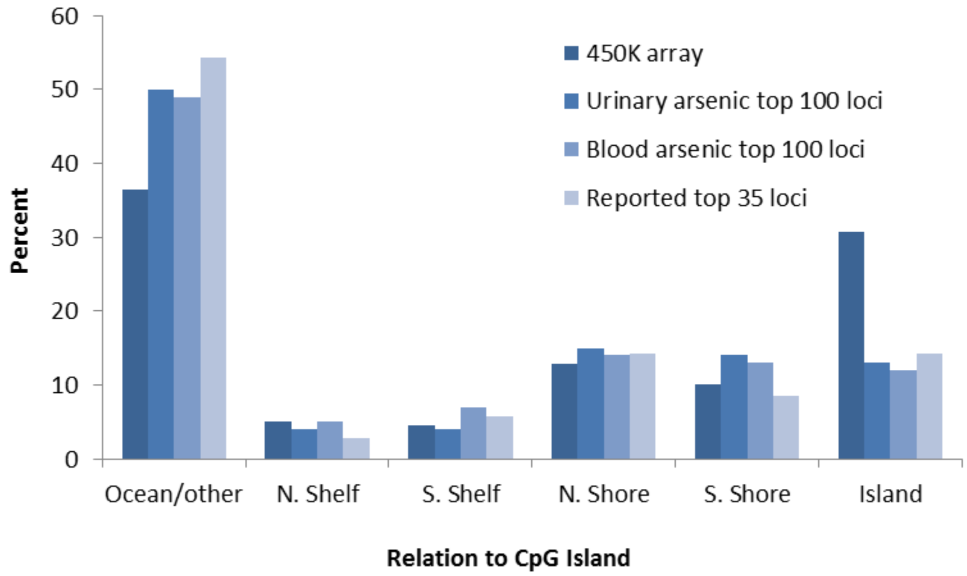

Figure S1. Distribution of methylation probes in relation to CpG functional regions for the 450K array, top 100 differentially methylated loci in relation to blood and urinary total arsenic concentrations, as well as reported top 35 loci. The distribution of CpG functional regions for the top 100 differentially methylated loci in relation to urinary total (P=0.0038) and blood (P=0.0030) arsenic concentrations were statistically different than the overall distribution of functional regions on the array. Funcational regions were classified as ocean/other (isolated CpG loci in the genome), shelf (2-4 kb from a CpG island), shore (within 2 kb of a CpG island), and island (region >200 bp in length and GC% >50%).

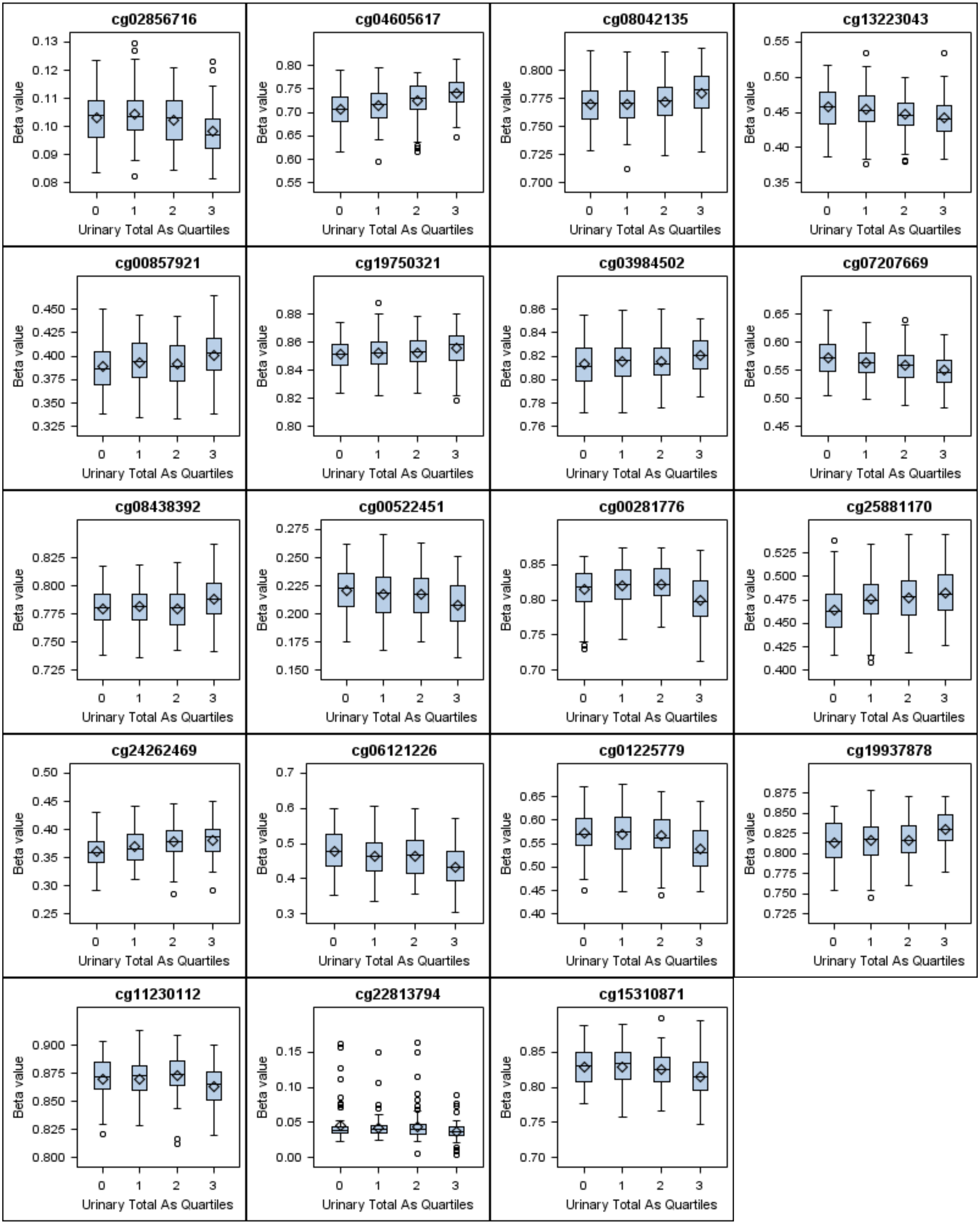

cg02856716
cg04605617
cg08042135
cg13223043
cg00857921
cg19750321
cg03984502
cg07207669
cg08438392
cg00522451
cg00281776
cg25881170
cg24262469
cg06121226
cg01225779
cg19937878
cg11230112
cg22813794
cg15310871
Beta value
Urinary Total As Quartiles
0
1
2
3

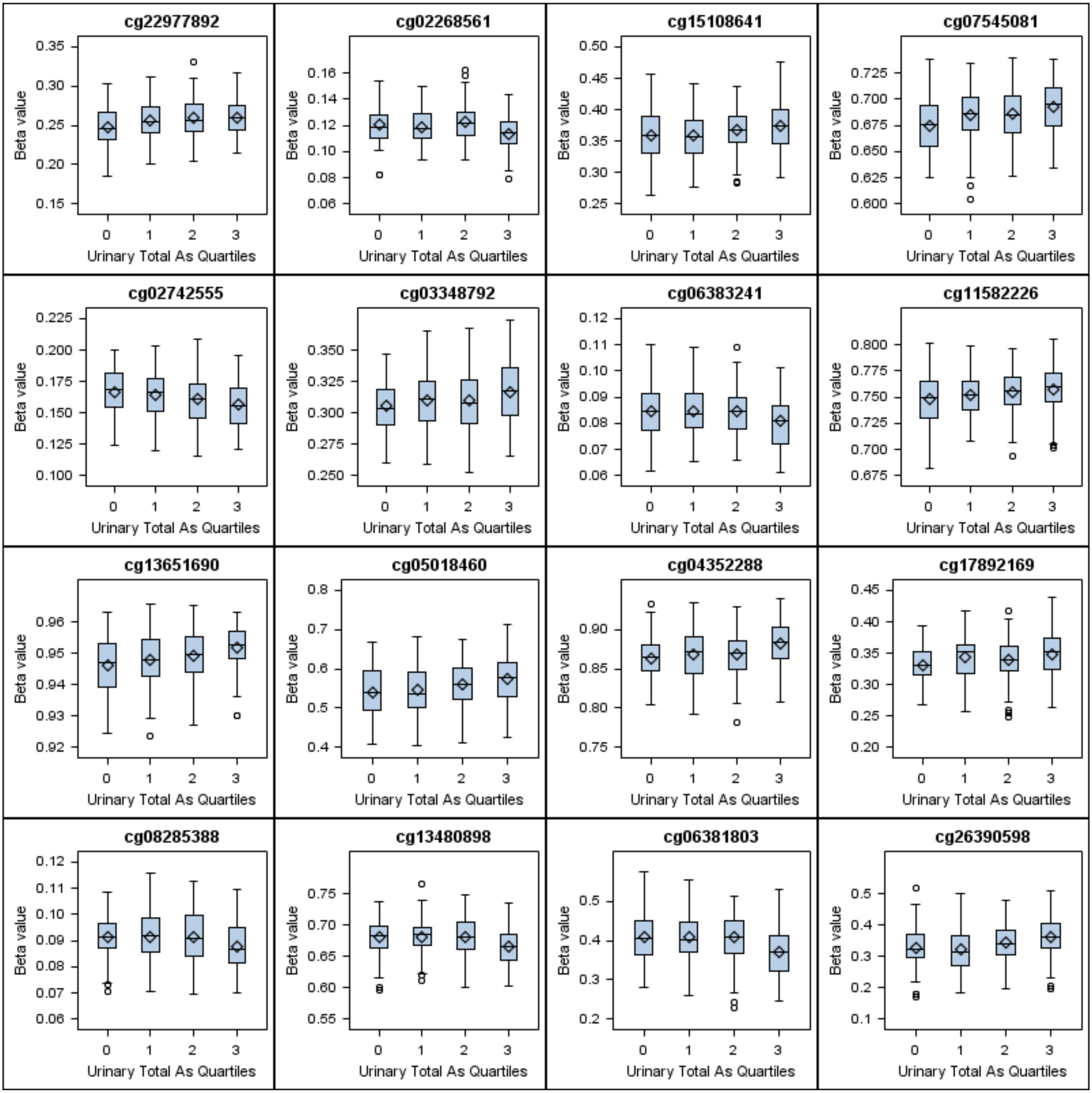

Figure S2. Boxplots of methylation β values by creatinine-adjusted urinary total arsenic concentration quartiles. The mean is depicted by the diamond, the median is depicted by the horizontal line in the box interior, the interquartile range (IQR) is represented by the length of the box, the lowest and highest data points within 1.5 IQR is depicted by the whiskers, and outlying values are depicted by the open circles.

Table S1. Lookup of 35 differentially methylation sites in arsenic-exposed replication sample.

Probe	Gene	P value	Low arsenic group average β value	High arsenic group average β value
cg13651690	*IGH*	0.0054*	0.932	0.945
cg02742555	*INSC*	0.0059*	0.145	0.128
cg24262469	*TIPARP-AS1*	0.0128*	0.237	0.216
cg07207669	*EFNA1*	0.0135*	0.550	0.529
cg17892169	*TNFSF12-TNFSF13*	0.0180*	0.251	0.232
cg03348792	*KRT1*	0.0287*	0.231	0.216
cg06381803	*EML2*	0.0327*	0.488	0.451
cg26390598	*B3GALT5*	0.0440*	0.311	0.337
cg11230112	*SYNJ2*	0.0745	0.910	0.919
cg00522451	*SLC20A1*||*NT5DC4*	0.0844	0.142	0.134
cg02856716	*PAX7*	0.0949	0.082	0.077
cg00857921	*TGFBR3*	0.1325	0.348	0.336
cg01225779	*SQSTM1*	0.1358	0.784	0.806
cg08285388	*DHRS13*	0.1540	0.074	0.071
cg11582226	*FBXL3*	0.1709	0.763	0.749
cg06383241	*MAP1LC3B2*	0.2035	0.059	0.057
cg22977892	*PPP2R2A*	0.2238	0.205	0.199
cg08042135	*PLA2G2C*	0.2323	0.756	0.764
cg08438392	*IFT172*	0.4044	0.738	0.731
cg02268561	*NMT2*	0.5154	0.113	0.111
cg13223043	*FAM110D*||*ZNF593*	0.5233	0.520	0.526
cg00281776	*PIKFYVE*||*PTH2R*	0.5740	0.892	0.893
cg07545081	*FAM53B*	0.5755	0.640	0.635
cg04352288	*CA5A*	0.7756	0.776	0.769
cg25881170	*CD47*	0.8251	0.464	0.464
cg04605617	*PLA2G2C*	0.8464	0.734	0.732
cg03984502	*RORC*	0.9052	0.750	0.746
cg19750321	*ARNT*	0.9205	0.850	0.847
cg13480898	*C19orf66*	0.9582	0.755	0.753
cg05018460	*AB240015*||*ARNT2*	NA		
cg06121226	*SLC4A4*	NA		
cg15108641	*UBTD1*	NA		
cg15310871	*ATP6V1B2*	NA		
cg19937878	*TBC1D7*	NA		
cg22813794	*MDH2*	NA		

NA indicates that data was not available after quality control processing for the probe in the replication dataset.

* P<0.05.

Table S2. Lookup of previously reported DNA methylation sites from epigenome-wide studies in relation to arsenic exposure or arsenical skin lesion status with P values reported from the Bangladesh dataset.

Methylation probe	P value - urinary As	P value - blood As
**Seow et al. 2014**		
cg11511175	0.0560	0.0458*
cg03333116	0.0770	0.0113*
cg27370573	0.0972	0.1476
cg03371700	0.1179	0.7077
cg17900076	0.1850	0.1112
cg00028056	0.3133	0.2947
cg01667892	0.3714	0.2730
cg19296405	0.3730	0.6214
cg25148456	0.4211	0.1370
cg10090568	0.4728	0.1994
cg00218770	0.4831	0.4419
cg24883732	0.5144	0.5834
cg02916425	0.6287	0.7410
cg24405174	0.6412	0.3057
cg06352616	0.6430	0.9336
cg02749948	0.6604	0.9000
cg07664579	0.6644	0.8212
cg22217449	0.7178	0.8952
cg03065888	0.7507	0.5124
cg15059639	0.7913	0.9954
**Kile et al. 2014**		
cg15641060	0.1705	0.2644
cg01783894	0.3637	0.1095
cg00122779	0.3655	0.2422
cg02095504	0.6517	0.5919
cg04597393	0.7367	0.4057
cg16321474	0.8562	0.6435
cg03783410	0.9325	0.8977
cg15346830	0.9563	0.8696
cg00498691	0.9637	0.5760
cg06448705	0.9730	0.4863
**Liu et al. 2014**		
cg22489759	0.0425*	0.0478*
cg00088989	0.0478*	0.0590
cg02276490	0.0741	0.0792
cg06722407	0.1598	0.1763
cg23417171	0.2255	0.0753
cg17428496	0.2452	0.1994
cg04690840	0.3277	0.2535
cg10389982	0.4854	0.4721
cg23997365	0.4978	0.5059

Methylation probe	P value - urinary As	P value - blood As
cg13611173	0.5054	0.5399
cg02824793	0.5713	0.8318
cg16793757	0.6398	0.7571
cg07604512	0.6877	0.9465
cg05622915	0.7005	0.6793
cg01040960	0.7181	0.4793
cg20682143	0.7809	0.2525
cg13311357	0.8193	0.9853
cg06696800	0.8558	0.7244
cg10014338	0.9419	0.7695
cg07175191	0.9609	0.8908
cg05651282	0.9821	0.7704
cg25417223	-	
**Yang et al. 2014**		
cg27504805	0.0529	0.0625
cg10887021	0.0781	0.2246
cg01936270	0.2104	0.3252
cg16306115	0.2294	0.0891
cg19953406	0.3312	0.5677
cg13877915	0.3781	0.1402
cg08315770	0.4447	0.4901
cg20723355	0.6429	0.7251
cg16319578	0.7848	0.5679
cg07748540	0.9573	0.9945
cg08107272	-	
**Koestler et al. 2014**		
cg03512414	0.0022*	0.0228*
cg11293029	0.0049*	0.0168*
cg09051215	0.0126*	0.0085*
cg01717164	0.0418*	0.0927
cg14993464	0.0818	0.1659
cg10729419	0.1372	0.4201
cg03840511	0.1589	0.4592
cg08120511	0.1632	0.1136
cg01410578	0.1935	0.1422
cg11793106	0.2486	0.2766
cg04248979	0.3137	0.4418
cg16817180	0.3139	0.7668
cg27514608	0.3165	0.9913
cg08884395	0.3558	0.6197
cg03887163	0.3918	0.7125
cg12117986	0.4062	0.4212
cg17519101	0.4560	0.3751
cg15156029	0.5481	0.5601
cg18003231	0.5547	0.6207
cg09417038	0.5834	0.7371

Methylation probe	P value - urinary As	P value - blood As
cg15091868	0.6461	0.7908
cg18151270	0.6819	0.9562
cg18535167	0.7036	0.8198
cg19270089	0.8101	0.9213
cg12513505	0.8283	0.8268
cg19213675	0.8655	0.8350
cg14185717	0.9611	0.7834
cg13495373	0.9703	0.6504
cg05423713	0.9789	0.5479
cg15206445	0.9997	0.4463

* $P<0.05$.

Table S3. Lookup of previously reported DNA methylation sites from candidate studies in relation to arsenic exposure with P values reported from the Bangladesh dataset.

Gene, methylation probe	P value - urinary As	P value - blood As
DAPK1		
cg17090012	0.0370*	0.0979
cg02100497	0.1908	0.1420
cg13778339	0.2537	0.1461
cg13805297	0.2147	0.1702
cg22571217	0.3668	0.2224
cg14134019	0.1571	0.2434
cg14159523	0.4690	0.3148
cg17984409	0.3122	0.3234
cg05475556	0.5625	0.3677
cg14089032	0.8991	0.4448
cg14071249	0.8467	0.4746
cg24754277	0.4686	0.4959
cg13765778	0.5448	0.5007
cg08797471	0.2886	0.5015
cg13814950	0.9827	0.5801
cg01463032	0.7796	0.5899
cg14286732	0.6062	0.6924
cg15746719	0.7287	0.7231
cg19734228	0.6378	0.7364
cg08719486	0.6077	0.7566
cg14014720	0.5874	0.8404
cg13527872	0.3511	0.8854
cg13823120	0.7872	0.9310
cg20401521	0.7632	0.9609
cg13964439	0.9312	0.9644
cg13932603	0.9672	0.9870
cg14250336	0.8171	0.9904
cg13752933	0.5810	0.9914
cg21180703	0.7910	0.9924
***CDKN2A* (*P16*)**		
cg03079681	0.0008*	0.0132*
cg04026675	0.2179	0.2687
cg12840719	0.9100	0.6441
cg13601799	0.9675	0.9298
cg07562918	0.7633	0.9833
GMDS		
cg11608390	0.0806	0.0366*
cg21499610	0.2235	0.0396*
cg01813738	0.0716	0.0479*
cg02145137	0.0595	0.0481*
cg25049733	0.0528	0.0498*
cg12303981	0.0792	0.0672
cg08804626	0.1328	0.0943

Gene, methylation probe	P value - urinary As	P value - blood As
cg17346428	0.2046	0.0965
cg00971613	0.1595	0.1026
cg09571972	0.0650	0.1162
cg18132076	0.0516	0.1196
cg19466906	0.0860	0.1324
cg12872692	0.2460	0.1390
cg07714812	0.1390	0.1633
cg07515565	0.2734	0.1829
cg09510246	0.7725	0.1882
cg21500247	0.1936	0.2258
cg20203534	0.1300	0.2355
cg16762849	0.2874	0.2412
cg17697739	0.3951	0.2501
cg26272879	0.5636	0.2827
cg17032507	0.4107	0.2851
cg04661959	0.5379	0.2987
cg11786839	0.0889	0.3093
cg22994582	0.1579	0.3324
cg19295314	0.3142	0.3337
cg06960457	0.3607	0.3433
cg01588725	0.8239	0.3436
cg09376855	0.7196	0.3545
cg14393923	0.1079	0.3565
cg09974772	0.7716	0.3570
cg08513472	0.8242	0.3702
cg26807095	0.2414	0.3723
cg06080793	0.7251	0.3805
cg25697442	0.7983	0.4033
cg10716548	0.8507	0.4095
cg07972026	0.5995	0.4377
cg13394216	0.6946	0.4445
cg27205941	0.8890	0.4517
cg01708377	0.2027	0.4637
cg27215185	0.9251	0.4707
cg12734024	0.1807	0.4934
cg01496600	0.7429	0.5004
cg25320587	0.9907	0.5035
cg03118021	0.0770	0.5636
cg20384898	0.3633	0.5671
cg21478123	0.2060	0.5672
cg11276500	0.4373	0.5855
cg19828338	0.2774	0.5892
cg27095915	0.8543	0.6004
cg23229309	0.9680	0.6213
cg21682057	0.7104	0.6258
cg14358088	0.8666	0.6577

Gene, methylation probe	P value - urinary As	P value - blood As
cg03878537	0.1847	0.6615
cg01588060	0.7129	0.6801
cg27478313	0.9544	0.6846
cg17305617	0.3710	0.6891
cg00741954	0.5353	0.6950
cg18434348	0.2938	0.7087
cg10707788	0.9631	0.7098
cg23120243	0.4949	0.7125
cg01465824	0.3533	0.7131
cg05996341	0.5491	0.7245
cg02926797	0.9385	0.7344
cg07329532	0.3384	0.7354
cg18551822	0.9435	0.7581
cg02783314	0.7033	0.7610
cg27294279	0.8620	0.7622
cg08614769	0.5232	0.8011
cg04695233	0.6279	0.8041
cg09841915	0.7446	0.8179
cg00750225	0.9950	0.8270
cg08932320	0.8898	0.8318
cg06315217	0.7806	0.8399
cg21347053	0.7516	0.8410
cg04497116	0.9329	0.8671
cg25936567	0.6254	0.8698
cg13253856	0.7632	0.8832
cg23411150	0.6291	0.8876
cg26546884	0.5457	0.8936
cg09953908	0.4760	0.9087
cg26908825	0.8852	0.9142
cg03058444	0.8445	0.9384
cg06007850	0.7844	0.9650
cg16550324	0.8590	0.9860
cg15193215	0.9744	0.9990
cg16137006	0.9607	0.9998
C10ORF32/AS3MT		
cg26626287	0.0062*	0.0348*
cg08330031	0.0634	0.0591
cg03769523	0.1797	0.1436
cg03275084	0.2123	0.1478
cg05959392	0.2507	0.1871
cg18534077	0.3536	0.2923
cg25193742	0.4954	0.3000
cg11050480	0.5150	0.3335
cg16330755	0.3420	0.4330
cg15744005	0.0470*	0.4642
cg26741530	0.3764	0.4689

Gene, methylation probe	P value - urinary As	P value - blood As
cg00086670	0.5911	0.4876
cg08668676	0.6251	0.5003
cg25729957	0.7095	0.5020
cg26207201	0.4438	0.5330
cg10615065	0.6967	0.5407
cg08757448	0.3751	0.5819
cg16989281	0.9161	0.6056
cg11784071	0.9523	0.6300
cg17259809	0.8659	0.6629
cg08856941	0.6048	0.6944
cg24592962	0.7522	0.7134
cg17255062	0.8004	0.7205
cg14557236	0.8281	0.8502
cg25143771	0.7107	0.8841
cg02921697	0.8685	0.8976
cg08772003	0.3159	0.9783
RASSF1		
cg00980904	0.1300	0.0672
cg12966367	0.1085	0.0754
cg13257331	0.0846	0.0770
cg03297783	0.3578	0.0836
cg11035216	0.2434	0.1088
cg22090713	0.1255	0.1126
cg21372200	0.1556	0.1126
cg25747192	0.1808	0.1184
cg14884256	0.2169	0.1226
cg08047457	0.4254	0.1269
cg15426878	0.5147	0.1651
cg13872831	0.1153	0.1863
cg24049629	0.0744	0.2016
cg00743929	0.2351	0.2329
cg13497155	0.3221	0.2769
cg21522636	0.0925	0.2797
cg05885095	0.1271	0.2964
cg21554552	0.2316	0.3047
cg06980053	0.0557	0.3113
cg06117233	0.0434*	0.3359
cg07130266	0.7208	0.3499
cg02930432	0.2504	0.3762
cg23147362	0.3339	0.3791
cg08078366	0.3144	0.3941
cg20826201	0.2648	0.4280
cg00777121	0.3784	0.4358
cg26093954	0.2836	0.4612
cg20119308	0.4708	0.4628
cg21418575	0.8372	0.4640

Gene, methylation probe	P value - urinary As	P value - blood As
cg09386807	0.7694	0.4648
cg19854901	0.1657	0.4862
cg19811994	0.6005	0.4903
cg06174454	0.4935	0.4946
cg26357744	0.6160	0.4973
cg10152523	0.7893	0.5047
cg06063729	0.5215	0.5054
cg04743654	0.1130	0.5095
cg24298409	0.8736	0.5218
cg01932734	0.4221	0.5265
cg19152024	0.7913	0.5354
cg24859722	0.2440	0.5443
cg27569446	0.6821	0.5876
cg04540383	0.7993	0.5912
cg21908110	0.3537	0.5916
cg06375085	0.5298	0.5966
cg06821120	0.6849	0.6007
cg05989693	0.6200	0.6081
cg22796393	0.4702	0.6136
cg06172942	0.4831	0.6666
cg27149285	0.8325	0.7480
cg02817007	0.9225	0.8186
cg01568433	0.7875	0.9121
cg07344955	0.5774	0.9165
cg15043975	0.7402	0.9616
cg25486143	0.6806	0.9707
cg05546296	0.9135	0.9918
PPARG		
cg10499651	0.1310	0.0257*
cg25929976	0.1009	0.1020
cg18063278	0.3556	0.1674
cg01412654	0.1835	0.1846
cg16827534	0.2376	0.2556
cg07556134	0.7498	0.3041
cg04908300	0.3904	0.3120
cg18537222	0.4706	0.4162
cg21859053	0.3835	0.4882
cg13518792	0.5413	0.4943
cg09405169	0.2839	0.5445
cg04632671	0.4008	0.5617
cg18887186	0.8130	0.5836
cg21946299	0.6694	0.6093
cg07676920	0.7375	0.6477
cg07895576	0.9847	0.6684
cg23514324	0.7392	0.6848
cg15722404	0.6541	0.7118

Gene, methylation probe	P value - urinary As	P value - blood As
cg04748988	0.6119	0.7519
cg27095527	0.7051	0.8231
cg06573644	0.8127	0.8608
cg16197186	0.9182	0.8716
cg26364899	0.7196	0.9826
TP53		
cg05479194	0.0159*	0.0041*
cg02855142	0.1444	0.0380*
cg08119584	0.1719	0.0715
cg01620719	0.0302*	0.0880
cg14351634	0.3578	0.0976
cg12041075	0.2695	0.1063
cg18198734	0.2135	0.1504
cg23290969	0.6054	0.1553
cg07760161	0.2769	0.2476
cg17461511	0.2342	0.2820
cg08691422	0.5861	0.4257
cg10792831	0.3479	0.4390
cg12041429	0.7084	0.4622
cg02087342	0.8645	0.4877
cg13169780	0.5220	0.5195
cg25896754	0.4858	0.5268
cg18311066	0.3047	0.5419
cg21050342	0.3151	0.5576
cg22175811	0.7993	0.5596
cg00807143	0.9929	0.5612
cg02842899	0.5454	0.6102
cg02690969	0.4763	0.6109
cg23846713	0.5634	0.6233
cg25053252	0.5613	0.6339
cg07991600	0.9171	0.6478
cg02045224	0.4224	0.6671
cg05348746	0.9374	0.6732
cg15206330	0.9310	0.6806
cg13468400	0.3761	0.6845
cg22949073	0.9418	0.6931
cg04009932	0.6909	0.7016
cg12373934	0.5787	0.7976
cg26539663	0.8796	0.8186
cg16397722	0.7196	0.8489
cg06317056	0.6082	0.8640
cg02166782	0.7802	0.9041
cg16203911	0.6957	0.9307
cg06365412	0.6289	0.9528
cg06587969	0.6854	0.9997

Gene, methylation probe	P value - urinary As	P value - blood As
MHL1		
cg11291081	0.0078*	0.0451*
cg04777024	0.0348*	0.0478*
cg05670953	0.0041*	0.0540
cg12851504	0.4298	0.0652
cg13846866	0.1836	0.0914
cg10769891	0.2819	0.1402
cg19132762	0.1321	0.1459
cg17024523	0.0749	0.1473
cg05845319	0.2701	0.1596
cg11600697	0.2947	0.2026
cg01302270	0.2725	0.2052
cg10990993	0.7643	0.2168
cg27586588	0.4999	0.2330
cg16433211	0.8920	0.2390
cg14671526	0.6122	0.2890
cg05906740	0.4453	0.2945
cg11363877	0.4166	0.3540
cg14598950	0.4289	0.3762
cg24607398	0.8110	0.4007
cg17641046	0.7081	0.4141
cg14751544	0.7281	0.4238
cg02279071	0.9145	0.4251
cg21109167	0.1755	0.4293
cg16764580	0.6471	0.4360
cg03497419	0.3112	0.4594
cg27331401	0.9758	0.4791
cg00893636	0.4995	0.5158
cg03192963	0.6067	0.5474
cg23658326	0.7797	0.5515
cg25837710	0.2331	0.5745
cg03405026	0.6626	0.5762
cg11224603	0.7240	0.6162
cg21490561	0.5565	0.6211
cg07064226	0.7734	0.6725
cg16863190	0.8385	0.6744
cg25202636	0.4074	0.6747
cg25212762	0.6925	0.6984
cg07101782	0.9040	0.7073
cg02103401	0.8289	0.7500
cg06791151	0.3299	0.7509
cg06108510	0.8743	0.7683
cg24985459	0.8298	0.7955
cg18320188	0.3373	0.8303
cg04726821	0.3231	0.8356
cg04841293	0.8882	0.8640

Gene, methylation probe	P value - urinary As	P value - blood As
cg17621259	0.8063	0.9192
cg06590608	0.9778	0.9195
cg19208331	0.7827	0.9366
cg12790037	0.7714	0.9387
cg03901257	0.8891	0.9540

*P<0.05.

References

Kile ML, Houseman EA, Baccarelli A, Quamruzzaman Q, Rahman M, Mostofa G, et al. 2014. Effect of prenatal arsenic exposure on DNA methylation and leukocyte subpopulations in cord blood. Epigenetics 9:774-782.

Koestler DC, Avissar-Whiting M, Houseman EA, Karagas MR, Marsit CJ. 2013a. Differential DNA methylation in umbilical cord blood of infants exposed to low levels of arsenic in utero. Environ Health Perspect 121:971-977.

Liu X, Zheng Y, Zhang W, Zhang X, Lioyd-Jones DM, Baccarelli AA, et al. 2014. Blood methylomics in response to arsenic exposure in a low-exposed us population. J Expo Sci Environ Epidemiol 24:145-149.

Seow WJ, Kile ML, Baccarelli AA, Pan WC, Byun HM, Mostofa G, et al. 2014. Epigenome-wide DNA methylation changes with development of arsenic-induced skin lesions in Bangladesh: a case-control follow-up study. Environ Mol Mutagen 55:449-456.

Yang TY, Hsu LI, Chiu AW, Pu YS, Wang SH, Liao YT, et al. 2014. Comparison of genome-wide DNA methylation in urothelial carcinomas of patients with and without arsenic exposure. Environ Res 128:57-63.
